# A Patient-Centered Emergency Department Management Strategy for Sickle-Cell Disease Super-Utilizers

**DOI:** 10.5811/westjem.2016.11.32273

**Published:** 2017-02-07

**Authors:** Grant G. Simpson, Hallie R. Hahn, Alex A. Powel, Robert R. Leverence, Linda A. Morris, Lara G. Thompson, Marc S. Zumberg, Deepa J. Borde, Joseph A. Tyndall, Jonathan J. Shuster, Donald M. Yealy, Brandon R. Allen

**Affiliations:** * University of Florida, Department of Pharmacology, Gainesville, Florida; † University of Florida, Department of Emergency Medicine, Gainesville, Florida; ‡ University of Florida Health, Department of Medicine, Division of Hospital; § Medicine, Gainesville, Florida; ¶ University of Florida, Department of Hematology/Oncology, Gainesville, Florida; || University of Florida Health, Care One Clinic, Division of Hospital Medicine, Gainesville, Florida; # University of Florida Health, Department of Health Outcomes and Policy, Gainesville, Florida; ** University of Pittsburgh and UPMC, Department of Emergency Medicine, Pittsburgh, Pennsylvania

## Abstract

**Introduction:**

A subpopulation of sickle-cell disease patients, termed super-utilizers, presents frequently to emergency departments (EDs) for vaso-occlusive events and may consume disproportionate resources without broader health benefit. To address the healthcare needs of this vulnerable patient population, we piloted a multidisciplinary intervention seeking to create and use individualized patient care plans that alter utilization through coordinated care. Our goals were to assess feasibility primarily, and to assess resource use secondarily.

**Methods:**

We evaluated the effects of a single-site interventional study targeted at a population of adult sickle-cell disease super-utilizers using a pre- and post-implementation design. The pre-intervention period was 06/01/13 to 12/31/13 (seven months) and the post-intervention period was 01/01/14 to 02/28/15 (14 months). Our approach included patient-specific best practice advisories (BPA); an ED management protocol; and formation of a “medical home” for these patients.

**Results:**

For 10 subjects targeted initially we developed and implemented coordinated care plans; after deployment, we observed a tendency toward reduction in ED and inpatient utilization across all measured indices. Between the annualized pre- and post-implementation periods we found the following: ED visits decreased by 16.5 visits/pt-yr (95% confidence interval [CI] [−1.32–34.2]); ED length of state (LOS) decreased by 115.3 hours/pt-yr (95% CI [−82.9–313.5]); in-patient admissions decreased by 4.20 admissions/pt-yr (95% CI [−1.73–10.1]); in-patient LOS decreased by 35.8 hours/pt-yr (95% CI [−74.9–146.7]); and visits where the patient left before treatment were reduced by an annualized total of 13.7 visits. We observed no patient mortality in our 10 subjects, and no patient required admission to the intensive care unit 72 hours following discharge.

**Conclusion:**

This effort suggests that a targeted approach is both feasible and potentially effective, laying a foundation for broader study.

## INTRODUCTION

The most common manifestation of sickle-cell disease in the emergency department (ED) is painful vaso-occlusive events.^1^–^3^ Many sickle-cell disease patients manage pain at home; some seek ED care for complications, infection, or most commonly a need for enhanced analgesia. Importantly, a small subpopulation of sickle-cell disease patients, termed super-utilizers, presents to EDs much more frequently than other patients with sickle-cell disease or variants.^4^ Approximately 20% of the sickle-cell disease patients account for more than half of ED visits by patients with this disease.^5^,^6^ This latter group may have more severe disease, less social support, consume more healthcare resources, and/or have an opportunity to better manage their care.^4^,^7^,^8^

A lack of coordinated care increases the frequency of unscheduled requests for medical care needs, particularly in EDs.^1^,^3^ Creating a management protocol along with enhanced support reduces ED and hospital utilization by sickle-cell disease patients.^9^ To date, no prior studies have evaluated a multidisciplinary approach that incorporates an ED protocol targeted specifically at super-utilizers.

We sought to test the development and introduction of a patient-centered management strategy targeting super-utilizer sickle-cell disease patients who presented to the ED with uncomplicated painful presentations. We wished to assess feasibility and preliminary impact on care measures.

## METHODS

### Study Design

We evaluated the effects of a single-site interventional study targeting a subset of adult sickle-cell disease patients, i.e. super-utilizers, using a pre- and post-implementation design. The pre-intervention period was 06/01/13 to 12/31/13 (seven months) and the post-intervention period was 01/01/14 to 02/28/15 (14 months). Our approach included patient-specific best practice advisories (BPA); an ED management protocol (Figure) with team-approved standing orders; and referral to a “medical home.” The institutional review board (IRB201500216) approved the retrospective analysis of medical records with a waiver of informed consent as no intervention was outside of common practice, though often variable. Therefore, this study did not use blinding.

### Study Setting and Population

Our study site was University of Florida, a Level I trauma and academic center in Gainesville, Florida, with an ED census of approximately 70,000 annual visits. We defined sickle-cell disease super-utilizers as adults (≥18 years of age) diagnosed with sickle-cell disease who presented to the ED 12 or more times in an average 12-month period.^6^

### Study Protocol

#### Intervention Development

Our interdisciplinary sickle-cell committee was formed in April 2013 and included community advocates and leaders from the local chapter of the Sickle Cell Disease Association, along with representatives from emergency medicine, internal medicine, hematology, pharmacy, ED and inpatient nursing, social work, psychology, and addiction psychiatry. The themes emerging during committee meetings were patient and provider frustration concerning lack of continuity of care, inconsistent treatment regimens, and reasons for elevated ED utilization. The team met monthly, inviting patients on occasion, and designed individualized care plans for super-utilizing patients.

#### Best Practice Advisories

We entered patient-specific care plans created by the multidisciplinary care team into the electronic medical record as a BPA so that all providers and staff would have immediate “pushed” access to the plan upon opening the patient’s chart. This BPA directed the provider to current recommendations from the interdisciplinary committee on pain management including patient-controlled analgesia settings, prior sickle-cell emergencies (e.g., acute chest syndrome, priapism), behavioral issues, and transfusion history. We revised plans regularly with input from the multidisciplinary team and continuous communication with hematology.

#### ED Protocol

The ED protocol for super-utilizers (Figure) helped guide decision-making, reduced redundant resource utilization, and standardized and expedited care. The multidisciplinary team created standing orders for pain control, imaging, and supportive care, which were implemented during the pre- and post-intervention periods to expedite care. We educated every ED resident and ED nurse via physician and nurse champions. A process improvement team formed and met monthly.

#### Medical Home

Study patients were referred to the “Care One Clinic,” a multidisciplinary hospital-based clinic for vulnerable patients with high-frequency ED visits. Upon enrollment, patients were seen by a primary care doctor, social worker and pharmacist, and had access to an embedded addiction and pain specialist and clinical psychologist. Pill counts, random drug screening, and self-documented pain scores were monitored closely. Missed appointments or aberrant behavior risked the cessation of analgesic prescriptions or institution of more frequent and stringent monitoring.

### Measures

We measured use of a plan, along with annualized frequency of ED visits; ED length of stay (LOS), measured from arrival to departure; frequency of admission; inpatient LOS; and left before treatment frequency. We also assessed 72-hour death from any cause and intensive care unit (ICU) admission after discharge as crude initial safety estimates.

### Data Analysis

We collected an aggregate de-identified dataset from the Decision Support Services data repository during the IRB-approved study period. We analyzed data using SAS Enterprise Guide version 9.4 (SAS Institute). We annualized study variables (i.e., multiple by 12 months/“n” months study period) for comparison. We primarily targeted descriptive measures given the feasibility and preliminary nature of the work, using 95% confidence intervals (CI) to assess those differences.

## RESULTS

Ten patients (five women) had an ED-based care plan developed. We confirmed use of BPAs and the ED protocol in all patients during ED visits. Overall, we found a tendency towards a decrease in all indices of emergency resource utilization. No patient mortality or ICU admissions 72 hours following discharge were observed.

### ED visits

We observed an (annualized) mean of 38.4 visits per patient per year (“pt-yr”) (standard deviation [SD]=23.9) in the pre-implementation period and 21.9 visits/pt-yr (SD=12.7) in the post-implementation period, representing a decrease of 16.5 visits/pt-yr (95% CI [−1.32, 34.2]) after implementation.

### ED LOS

The super-utilizers were seen in the ED for a mean of 305.3 hours/pt-yr (SD=247.4) in the pre-implementation period and 190.0 hours/pt-yr (SD=160.9) in the post-implementation period, representing a decrease of 115.3 hours/pt-yr (95% CI [−82.9–313.5] after implementation.

### In-patient admissions

We observed a mean 12.2 admissions/pt-yr (SD=3.23) in the pre-implementation period and 7.97 admissions/pt-yr (SD=7.78) in the post-implementation period, representing a decrease of 4.20 admissions/pt-yr (95% CI [−1.73–10.1) after implementation.

### In-patient LOS

Those super-utilizers admitted had an in-patient mean LOS of 142.2 hours/pt-yr (SD=147.7) in the pre-implementation period and 106.3 hours/pt-yr (SD=127.5) in the post-implementation period, representing a decrease of 35.9 hours/pt-yr (95% CI [−74.9–146.7]) after implementation.

### Left Before Treatment

The total annualized number of patient visits in which the patient left without being seen or left against medical advice (LWBS/AMA) was 20.6 in pre-implementation, 6.85 in post-implementation, representing a reduction of 13.7 visits.

## DISCUSSION

Sickle-cell disease patients who more frequently present to the ED are often more severely ill as indicated by laboratory values, report greater pain, and have more complications from their condition than the standard sickle-cell disease healthcare user.^4^,^7^ Repeated admissions for pain control correlate with higher mortality rates.^8^ The chronicity and rapidity of these pain episodes reduce sickle-cell disease patients’ quality of life.^4^,^6^,^9^ Because sickle-cell disease predominantly affects minority populations, the manifestations of the disease exacerbate the challenges faced by communities with high proportions of minority residents.^10^ This motivated us to develop a protocol of care in an effort to improve the medical care provided to the sickle-cell disease population at our institution. While research on standard sickle-cell disease patient care exists, little is known about very high care utilizers, the population we targeted. Koch reported an intensive management strategy centered on opening a designated sickle-cell disease day hospital, which led to reductions in healthcare use in their high-utilizer and super-utilizer groups.^6^ We studied exclusively super-utilizers using a multidisciplinary, ED-based strategy and found similar effects. This suggests that implementing similar protocols at other academic and community institutions is both possible and potentially effective in achieving reductions in resource utilization. Further, we observed that none of our patients required ICU care 72 hours following a discharge, nor did we observe any patient mortality.

Previously published studies highlight the viability of patient-centered management strategies.^6^,^9^ Our patient-centered multidisciplinary care team and the inclusion of advocates for sickle-cell disease patients fostered relationships between the hospital and community. Reduction in LWBS/AMA rates suggests an expectation of improved care as a result of the individualized care plans.

## LIMITATIONS

Our trial was primarily a feasibility effort and not designed or powered to detect specific differences; while we saw lower resource use across all who had a plan enacted, the small sample does not allow us to quantify a durable magnitude effect but does offer promise for future work in other settings. We cannot perform a granular assessment of safety due to our small sample, though our current short-term signal was not negative. We did not measure patient satisfaction, though anecdotally the acceptance and unstructured feedback were high among providers and patients. Overall costs measurement was not a focus, but the broad reduction in admission and other resource utilization will very likely translate into a cost savings with this approach.

The study was not blinded, i.e., the providers were aware of the interventions and the institution’s new standard of care, which may have improved results. The Hawthorne effect could also be influencing the patient outcomes we observed, which does limit interpretation of the results.

## CONCLUSION

An individualized care plan created by a multidisciplinary care team can be created and used in a population of sickle-cell disease super-ED users linked to a tendency to reduce healthcare utilization. The scalability and cost effectiveness of this approach is fertile ground for new research in this under-explored topic.

## Figures and Tables

**Figure:**
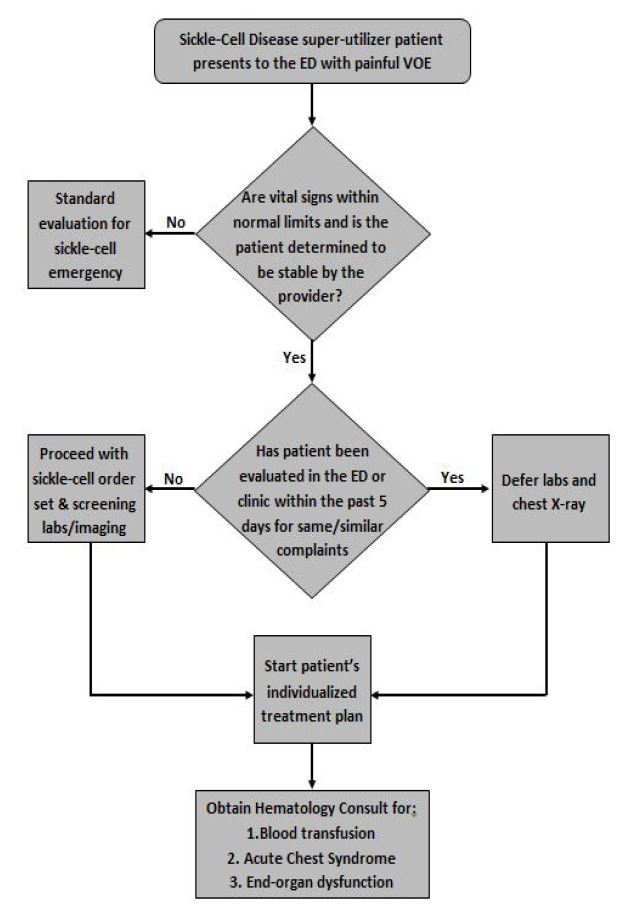
The sickle-cell disease super-utilizer protocol was designed to expedite analgesic administration and reduce redundant laboratory tests and imaging for patients meeting inclusion criteria. If patients are determined to be clinically stable by the provider with appropriate vital signs, the patient’s treatment plan developed by the multidisciplinary team can be implemented, thereby expediting and standardizing care. If the patient has been evaluated by laboratory examination or radiographs in the ED or clinic within five days of their current ED visit, the provider can defer work-up at that time and implement the patient’s treatment plan, serving to reduce redundant work-up and further expedite and standardize care. If a hematologic emergency is suspected, a comprehensive evaluation is warranted based on provider discretion. *ED*, emergency department; *VOE*, vaso-occlusive event.
